# *Synergus nigrus* (Hymenoptera, Cynipidae, Synergini), a new inquiline species reared from galls of *Philonix nigra* Gillette, 1889 (Cynipidae, Cynipini)

**DOI:** 10.3897/BDJ.14.e170671

**Published:** 2026-03-27

**Authors:** Denise C Montelongo, Louis F Nastasi, Heather M Hines, Andrew R Deans

**Affiliations:** 1 California Academy of Sciences, San Francisco, United States of America California Academy of Sciences San Francisco United States of America; 2 The Pennsylvania State University, University Park, United States of America The Pennsylvania State University University Park United States of America; 3 Department of Biology, The Pennsylvania State University, University Park, United States of America Department of Biology, The Pennsylvania State University University Park United States of America; 4 Frost Entomological Museum, The Pennsylvania State University, University Park, United States of America Frost Entomological Museum, The Pennsylvania State University University Park United States of America

**Keywords:** gall wasp, inquiline, COI

## Abstract

**Background:**

*Philonix nigra* Gillette, 1889 (Hymenoptera, Cynipidae, Cynipini) induces novel structures, plant galls, on white oaks (Fagaceae: *Quercus alba* L. and possibly other species classified in Quercus sect. Quercus). Such structures are often inhabited by other cynipids, who apparently cannot induce their own galls on these plants. These inquilinous species are primarily classified in the tribes Synergini (*Synergus* spp.) and Ceroptresini (*Ceroptres* spp.) Extensive rearing pf *P. nigra* galls has yielded numerous associates, including a new species of *Synergus*.

**New information:**

We describe *Synergus nigrus* Montelongo & Nastasi **sp. nov.**, a new species of inquilinous cynipid discovered from the eastern USA in *Philonix nigra* galls. This species is morphologically diagnosable, being the first *Synergus* with a partially smooth mesopleuron and 15 antennomeres in females. This diagnosis was also supported by DNA barcode data (mitochondrial cytochrome c oxidase I gene fragment), when compared to other available Synergini barcode sequences. We discuss key aspects that separate this species from related species and issues with taxonomy of this genus.

## Introduction

*Synergus* Hartig, 1840 is the most species-rich genus of inquilinous cynipids, with about 130 described species distributed throughout the Holarctic Region (Schwéger et al. 2015, Lobato-Vila et al. 2020b). *Synergus* can be separated from the rest of the inquiline tribe Synergini by five morphological characters (Schwéger et al. 2015). Nevertheless, recent studies have revealed *Synergus* to be a polyphyletic group, with Palaearctic species (*Synergus* *s.s.*) forming a distinct lineage relative to the Nearctic and Neotropical species and the New World species separated into three clades (*Synergus s.l.)* (Ward et al. 2020, Lobato-Vila et al. 2022); thus, a revised taxonomy and set of diagnostic traits is needed. Recent phylogenetic data suggest that several species of *Synergus* likely remain undescribed (Ward et al. 2020, Xiudan et al. 2024). Improved understanding of species diversity in this clade will provide information for the biology and host evolution in these inquiline wasps.

Recent studies have combined molecular data with morphological, ecological and phenology data to delimit species of *Synergus* (Ward et al. 2020, Xiudan et al. 2024). Ward et al. (2020) suggest that *Philonix nigra* Gillette 1889 galls likely host a new species of *Synergus*, which has not been described.

*Philonix nigra* induces galls on the leaves of *Quercus alba* L., *Q. bicolor* Willd., *Q. prinoides* Willd., *Q. macrocarpa* Michx. and *Q. muehlenbergii* Engelm. (Fagaceae) in eastern North America in the spring (Weld 1959). The galls abscise from leaf veins dropping to the forest floor when mature and the adult emerges in the winter (Weld 1922). In 2020, we conducted a study looking at the associated arthropods of the asexual generation of the gall-inducing wasp *P. nigra*, on *Q. alba* in central Pennsylvania (USA) featuring a broad range of topics from natural history, gall food webs and resource partitioning (Montelongo 2021; Montelongo et al. in prep.). Amongst the list of associated arthropods, an undescribed species of *Synergus* was found that aligns with that uncovered by Ward et al. (2020) and which is described herein.

## Materials and methods

### Gall sampling and rearing

*Synergus* adults were reared from *P. nigra* galls on *Q. alba* collected in Pennsylvania in 2020 from locations in State College (40.806, -77.913 and 40.801, -77.869) and Pine Grove Mills (40.731, -77.889). To ensure they experienced natural light/dark cycles and ambient temperatures and humidity, galls were separated and placed in individual souffle cups in a clear storage tote container outdoors under a roof. Containers were checked for emergence every 2 or 3 days and emergent insects were placed in 80% ethanol in 2 ml microtubes with a label containing the gall’s unique code and the emergence date. Exemplars of each morphospecies obtained during rearing were point-mounted for morphological observation. Thirteen *Synergus* females and seven *Synergus* males emerged during the study. Adults were identified as *Synergus* using the key in Zimmerman (2018) and confirmed with the following set of diagnostic traits: (1) presence of the lateral pronotal and frontal carinae; (2) females usually with 14 antennomeres (can have 13 or 15) and 15 for males; (3) marginal cell on fore wing usually closed; (4) clypeus indistinct; and (5) lower face covered with radiating striations (Schwéger et al. 2015).

### Imaging

We performed morphological observations of *S. nigrus* using an Olympus SZX16 stereomicroscope (Olympus Life Science, Tokyo, Japan) fitted with an optical micrometer. We measured antenna segments at a resolution of 0.005 (1/200) millimetres using 10× magnification in combination with the 2× objective. Other measurements were taken at an appropriate magnification using the 1× objective. Our lighting setup consisted of a gooseneck illuminator fitted with mylar strips to diffuse light and we used an additional mylar strip fashioned into a ring to further diffuse light around the specimen itself.

We imaged the holotype using a Macroscopic Solutions ‘microkit’ (Tolland, CT) imaging station. Photos were stacked using Zerene Stacker LLC (Richland, WA). We edited images and prepared plates using Adobe Photoshop and Adobe Illustrator (Adobe Inc.).

### Morphological terminology

We follow the Hymenoptera Anatomy Ontology (HAO) (Yoder et al. 2010) for morphological terminology, as documented in Suppl. material 2, following Seltmann et al. (2012). These morphological concepts are primarily derived from the following sources: adult structures from Liljeblad and Ronquist (2002), Melika (2006) and Lobato-Vila and Pujade-Villar (2021); fore wing venation from Ronquist and Nordlander (1989), and surface sculpture from Harris (1979).

### Molecular methods

We employed a DNA barcoding approach (Hebert et al. 2003) to compare six reared *Synergus* specimens (5 females and 1 male) of the new morphospecies to barcode sequences in known *Synergus* species. DNA was extracted from legs of dry-preserved specimens using the E.Z.N.A. MicroElute Genomic DNA Kit from Omega Bio-tek in accordance with the manufacturer’s protocol. A 643 base pair (bp) COI region was amplified using NEB Taq Mastermix and primers LEP-F1, 5′-ATTCAACCAATCATAAAGATAT-3′ and LEP-R1, 5′-TAAACTTCTGGATGTCCAAAAA-3′. The thermal cycling programme began with a 2-minute denaturing step at 94°C, followed by 5 cycles of 30 seconds at 94°C (denaturing), 40 s at 45°C (annealing) and 60 s at 72°C (extension), followed by 35 cycles containing an annealing temperature of 50°C, 10 minutes at 72°C and a standby temperature of 4°C. The resulting PCR products were cleaned using EXOSAP-IT and using standard protocols prior to single strand Sanger sequencing on an Applied Biosystems 3730XL in the Huck Institutes of the Life Sciences’ (Penn State). Geneious Prime (Biomatters Ltd., Auckland; version 2024.0.7) was used to edit the sequences.

To compare our morphospecies to existing *Synergus* and the rest of the genera in the tribe Synergini, we obtained the sequences (COI) utilised in the most recent Synergini phylogenetic tree (Lobato-Vila et al. 2022). We also included the following species sequences (COI) that were absent in the previous analysis: *Synergus laeviventris* (Osten-Sacken, 1891) (MT124934), *Synergus magnus* Gillette, 1891 (MT124920), *Synergus villosus* Gillette, 1891 (MT124931) and *Ufo nipponicus* Melika, 2012 (JX468361) (Melika et al. 2012, Ward et al. 2020). The sequences of *Rhoophilus loewi* Mayr, 1881 from Lobato-Vila et al. (2022) were included as the outgroup, due to the established position of Rhoophilini as sister to Synergini in said phylogenetic analyses. The six newly-obtained COI sequences were queried against all available nucleotide sequences in GenBank using BLASTN (Madden 2013). The strongest matches not blasting to sequences of species already included led us to add two sequences of unidentified individuals to our analysis: *Synergus* sp. #2 (MT124876) and Cynipidae sp. BIOUG31033-E06 (MG443838). A total of 114 specimens were utilised in our analysis (Suppl. material 1). We used MAFFT 7 to automatically align the sequences through its web server (Katoh and Standley 2013).

The aligned sequences from each species (658 bp of COI) were analysed with IQ-TREE 3.0.1 (Wong et al. 2025), using Maximum Likelihood (ML). The ML analysis was conducted using the edge-linked partition model (Chernomor et al. 2016) and best partition scheme followed by tree inference substitution model Kalyaanamoorthy et al. 2017), with 1,000 ultrafast bootstrap (UFBoot) approximation replicates (Minh et al. 2013) and 1,000 replicates of Shimodaira-Hasegawa approximate likelihood ratio test (SH-aLRT; Guindon et al. (2010)). The resulting tree was visualised with FigTree v.1.4.4 (https://github.com/rambaut/figtree/) and prepared as illustrations in Inkscape (v.1.4; https://inkscape.org/).

### Abbreviations

Abbreviations and terms used in text and figures:

- DLO: Diameter of lateral ocellus, largest possible diameter of either lateral ocellus;
- F1 and F2: First and second antennal flagellomeres;
- LOL: Lateral ocellar line, shortest distance between margins of median and lateral ocelli;
- OOL: Ocular ocellar line, shortest distance between inner orbit and outer margin of posterior ocellus;
- POL: Posterior ocellar line, shortest distance between inner margins of posterior ocelli.

Institutional abbreviations:

- INHS - Illinois Natural History Survey, Champaign, IL., USA;
- PSUC - Frost Entomological Museum, The Pennsylvania State University, University Park, PA, USA;
- USNM - United States National Museum, Smithsonian Institution, Washington, D.C., USA.

## Taxon treatments

### Synergus nigrus

Montelongo & Nastasi sp. nov.

9EF83F1A-CCAC-5CD7-8783-5EE1ED7E051C

0BDCF679-2C7C-42F2-9EF3-F2C62B07D71C

#### Materials

- **Type status:** Holotype. **Occurrence:** catalogNumber: PSUC_FEM_79450; occurrenceRemarks: emerged 30.vii-2.viii.2020 | Dropped Gall; recordedBy: Laura Porturas; individualCount: 1; sex: female; lifeStage: adult; occurrenceID: 2A28B080-570D-5BEA-B1EC-6BA677C86C52; **Taxon:** scientificName: Synergus nigrus; order: Hymenoptera; family: Cynipidae; **Location:** stateProvince: Pennsylvania; county: Centre; municipality: State College; locality: Park Forest Village Neighbourhood; decimalLatitude: 40.806; decimalLongitude: -77.913; geodeticDatum: WGS84; **Identification:** identifiedBy: Denise C. Montelongo; **Event:** samplingProtocol: hand collected; eventDate: 2020-07-07; fieldNumber: DCM2020-242; **Record Level:** institutionCode: PSUC; basisOfRecord: PreservedSpecimen
- **Type status:** Paratype. **Occurrence:** catalogNumber: PSUC_FEM_79451; occurrenceRemarks: emerged 25-27.vii.2020 | Dropped Gall; recordedBy: Laura Porturas; individualCount: 1; sex: female; lifeStage: adult; occurrenceID: 2F938F8B-BD7A-551D-A354-DD2154BA6C0A; **Taxon:** scientificName: Synergus nigrus; order: Hymenoptera; family: Cynipidae; **Location:** stateProvince: Pennsylvania; county: Centre; municipality: State College; locality: Park Forest Village Neighbourhood; decimalLatitude: 40.806; decimalLongitude: -77.913; geodeticDatum: WGS84; **Identification:** identifiedBy: Denise C. Montelongo; **Event:** samplingProtocol: hand collected; eventDate: 2020-07-07; fieldNumber: DCM2020-246; **Record Level:** institutionCode: PSUC; basisOfRecord: PreservedSpecimen
- **Type status:** Paratype. **Occurrence:** catalogNumber: PSUC_FEM_79452; occurrenceRemarks: emerged 27-30.vii.2020 | Dropped Gall; recordedBy: Laura Porturas; individualCount: 1; sex: female; lifeStage: adult; occurrenceID: 7546BCB6-A97E-550B-9C92-192627DDB401; **Taxon:** scientificName: Synergus nigrus; order: Hymenoptera; family: Cynipidae; **Location:** stateProvince: Pennsylvania; county: Centre; municipality: State College; locality: Park Forest Village Neighbourhood; decimalLatitude: 40.806; decimalLongitude: -77.913; geodeticDatum: WGS84; **Identification:** identifiedBy: Denise C. Montelongo; **Event:** samplingProtocol: hand collected; eventDate: 2020-07-07; fieldNumber: DCM2020-229; **Record Level:** institutionCode: PSUC; basisOfRecord: PreservedSpecimen
- **Type status:** Paratype. **Occurrence:** catalogNumber: PSUC_FEM_79453; occurrenceRemarks: emerged 30.vii-2.viii.2020 | Dropped Gall; recordedBy: Laura Porturas; individualCount: 1; sex: female; lifeStage: adult; occurrenceID: C30C1FBD-CC29-511C-A570-DF1834CD416F; **Taxon:** scientificName: Synergus nigrus; order: Hymenoptera; family: Cynipidae; **Location:** stateProvince: Pennsylvania; county: Centre; municipality: State College; locality: Park Forest Village Neighbourhood; decimalLatitude: 40.806; decimalLongitude: -77.913; geodeticDatum: WGS84; **Identification:** identifiedBy: Denise C. Montelongo; **Event:** samplingProtocol: hand collected; eventDate: 2020-07-07; fieldNumber: DCM2020-247; **Record Level:** institutionCode: PSUC; basisOfRecord: PreservedSpecimen
- **Type status:** Paratype. **Occurrence:** catalogNumber: PSUC_FEM_79454; occurrenceRemarks: emerged 30.vii-2.viii.2020 | Dropped Gall; recordedBy: Laura Porturas; individualCount: 1; sex: female; lifeStage: adult; occurrenceID: 6609CF3B-555D-506F-8344-4ACD7EF20698; **Taxon:** scientificName: Synergus nigrus; order: Hymenoptera; family: Cynipidae; **Location:** stateProvince: Pennsylvania; county: Centre; municipality: State College; locality: Park Forest Village Neighbourhood; decimalLatitude: 40.806; decimalLongitude: -77.913; geodeticDatum: WGS84; **Identification:** identifiedBy: Denise C. Montelongo; **Event:** samplingProtocol: hand collected; eventDate: 2020-07-07; fieldNumber: DCM2020-239; **Record Level:** institutionCode: PSUC; basisOfRecord: PreservedSpecimen
- **Type status:** Paratype. **Occurrence:** catalogNumber: PSUC_FEM_79455; occurrenceRemarks: emerged 25-27.vii.2020 | Dropped Gall; recordedBy: Laura Porturas; individualCount: 1; sex: male; lifeStage: adult; occurrenceID: 685C7A8A-EC8F-5058-AC33-53232E3BB0F0; **Taxon:** scientificName: Synergus nigrus; order: Hymenoptera; family: Cynipidae; **Location:** stateProvince: Pennsylvania; county: Centre; municipality: State College; locality: Park Forest Village Neighbourhood; decimalLatitude: 40.806; decimalLongitude: -77.913; geodeticDatum: WGS84; **Identification:** identifiedBy: Denise C. Montelongo; **Event:** samplingProtocol: hand collected; eventDate: 2020-07-07; fieldNumber: DCM2020-241; **Record Level:** institutionCode: PSUC; basisOfRecord: PreservedSpecimen
- **Type status:** Paratype. **Occurrence:** catalogNumber: PSUC_FEM_79456; occurrenceRemarks: emerged 27-30.vii.2020 | Dropped Gall; recordedBy: Laura Porturas; individualCount: 1; sex: male; lifeStage: adult; occurrenceID: E69C5A09-9029-5A8F-9A63-FE73565147F9; **Taxon:** scientificName: Synergus nigrus; order: Hymenoptera; family: Cynipidae; **Location:** stateProvince: Pennsylvania; county: Centre; municipality: State College; locality: Park Forest Village Neighbourhood; decimalLatitude: 40.806; decimalLongitude: -77.913; geodeticDatum: WGS84; **Identification:** identifiedBy: Denise C. Montelongo; **Event:** samplingProtocol: hand collected; eventDate: 2020-07-07; fieldNumber: DCM2020-231; **Record Level:** institutionCode: PSUC; basisOfRecord: PreservedSpecimen
- **Type status:** Paratype. **Occurrence:** catalogNumber: PSUC_FEM_79457; occurrenceRemarks: emerged 27-30.vii.2020 | Dropped Gall; recordedBy: Laura Porturas; individualCount: 1; sex: male; lifeStage: adult; occurrenceID: 8B9ED023-1947-54DB-BDB8-94BE8BF2ACAB; **Taxon:** scientificName: Synergus nigrus; order: Hymenoptera; family: Cynipidae; **Location:** stateProvince: Pennsylvania; county: Centre; municipality: State College; locality: Park Forest Village Neighbourhood; decimalLatitude: 40.806; decimalLongitude: -77.913; geodeticDatum: WGS84; **Identification:** identifiedBy: Denise C. Montelongo; **Event:** samplingProtocol: hand collected; eventDate: 2020-07-07; fieldNumber: DCM2020-236; **Record Level:** institutionCode: PSUC; basisOfRecord: PreservedSpecimen
- **Type status:** Paratype. **Occurrence:** catalogNumber: PSUC_FEM_79458; occurrenceRemarks: emerged 2-4.viii.2020 | Dropped Gall; recordedBy: Andrew R. Deans; individualCount: 1; sex: male; lifeStage: adult; occurrenceID: B266E6BA-9068-5131-84A1-3BBD75F1B787; **Taxon:** scientificName: Synergus nigrus; order: Hymenoptera; family: Cynipidae; **Location:** stateProvince: Pennsylvania; county: Centre; municipality: Ferguson Township; locality: Pine Grove Mills; decimalLatitude: 40.731; decimalLongitude: -77.889; geodeticDatum: WGS84; **Identification:** identifiedBy: Denise C. Montelongo; **Event:** samplingProtocol: hand collected; eventDate: 2020-07-27; fieldNumber: DCM2020-358; **Record Level:** institutionCode: PSUC; basisOfRecord: PreservedSpecimen
- **Type status:** Paratype. **Occurrence:** catalogNumber: PSUC_FEM_79459; occurrenceRemarks: emerged 9-11.viii.2020 | Dropped Gall; recordedBy: Andrew R. Deans; individualCount: 1; sex: male; lifeStage: adult; occurrenceID: B364AEDE-48B7-5970-A1CA-13E5C3B3AA43; **Taxon:** scientificName: Synergus nigrus; order: Hymenoptera; family: Cynipidae; **Location:** stateProvince: Pennsylvania; county: Centre; municipality: Ferguson Township; locality: Pine Grove Mills; decimalLatitude: 40.731; decimalLongitude: -77.889; geodeticDatum: WGS84; **Identification:** identifiedBy: Denise C. Montelongo; **Event:** samplingProtocol: hand collected; eventDate: 2020-07-27; fieldNumber: DCM2020-394; **Record Level:** institutionCode: PSUC; basisOfRecord: PreservedSpecimen
- **Type status:** Paratype. **Occurrence:** catalogNumber: PSUC_FEM_79460; occurrenceRemarks: emerged 30.vii-2.viii.2020 | Dropped Gall; recordedBy: Laura Porturas; individualCount: 1; sex: female; lifeStage: adult; occurrenceID: DCA8F865-F559-57FB-AA15-3944191F18B0; **Taxon:** scientificName: Synergus nigrus; order: Hymenoptera; family: Cynipidae; **Location:** stateProvince: Pennsylvania; county: Centre; municipality: State College; locality: Park Forest Village Neighbourhood; decimalLatitude: 40.806; decimalLongitude: -77.913; geodeticDatum: WGS84; **Identification:** identifiedBy: Denise C. Montelongo; **Event:** samplingProtocol: hand collected; eventDate: 2020-07-07; fieldNumber: DCM2020-245; **Record Level:** institutionCode: INHS; basisOfRecord: PreservedSpecimen
- **Type status:** Paratype. **Occurrence:** catalogNumber: PSUC_FEM_79461; occurrenceRemarks: emerged 7-9.ix.2020 | Dropped Gall; recordedBy: Denise C. Montelongo; individualCount: 1; sex: female; lifeStage: adult; occurrenceID: C23436BB-81AE-5BA5-B5F8-162A9B59B621; **Taxon:** scientificName: Synergus nigrus; order: Hymenoptera; family: Cynipidae; **Location:** stateProvince: Pennsylvania; county: Centre; municipality: University Park; locality: Stuckeman Library at PSU; decimalLatitude: 40.801; decimalLongitude: -77.869; geodeticDatum: WGS84; **Identification:** identifiedBy: Denise C. Montelongo; **Event:** samplingProtocol: hand collected; eventDate: 2020-07-15; fieldNumber: DCM2020-560; **Record Level:** institutionCode: INHS; basisOfRecord: PreservedSpecimen
- **Type status:** Paratype. **Occurrence:** catalogNumber: PSUC_FEM_79462; occurrenceRemarks: emerged 27-30.vii.2020 | Dropped Gall; recordedBy: Laura Porturas; individualCount: 1; sex: male; lifeStage: adult; occurrenceID: FF0F5BCB-B128-5C6E-8116-916C9BD26C76; **Taxon:** scientificName: Synergus nigrus; order: Hymenoptera; family: Cynipidae; **Location:** stateProvince: Pennsylvania; county: Centre; municipality: State College; locality: Park Forest Village Neighbourhood; decimalLatitude: 40.806; decimalLongitude: -77.913; geodeticDatum: WGS84; **Identification:** identifiedBy: Denise C. Montelongo; **Event:** samplingProtocol: hand collected; eventDate: 2020-07-07; fieldNumber: DCM2020-238; **Record Level:** institutionCode: INHS; basisOfRecord: PreservedSpecimen
- **Type status:** Paratype. **Occurrence:** catalogNumber: PSUC_FEM_79463; occurrenceRemarks: emerged 27-30.vii.2020 | Dropped Gall; recordedBy: Laura Porturas; individualCount: 1; sex: female; lifeStage: adult; occurrenceID: 2CD73337-AEAF-5826-A3C6-423B2D4D3B45; **Taxon:** scientificName: Synergus nigrus; order: Hymenoptera; family: Cynipidae; **Location:** stateProvince: Pennsylvania; county: Centre; municipality: State College; locality: Park Forest Village Neighbourhood; decimalLatitude: 40.806; decimalLongitude: -77.913; geodeticDatum: WGS84; **Identification:** identifiedBy: Denise C. Montelongo; **Event:** samplingProtocol: hand collected; eventDate: 2020-07-07; fieldNumber: DCM2020-235; **Record Level:** institutionCode: INHS; basisOfRecord: PreservedSpecimen
- **Type status:** Paratype. **Occurrence:** catalogNumber: PSUC_FEM_79464; occurrenceRemarks: emerged 27-30.vii.2020 | Dropped Gall; recordedBy: Laura Porturas; individualCount: 1; sex: female; lifeStage: adult; occurrenceID: 9DD9BA20-D329-5A8E-AE26-5C28D8C1F4DB; **Taxon:** scientificName: Synergus nigrus; order: Hymenoptera; family: Cynipidae; **Location:** stateProvince: Pennsylvania; county: Centre; municipality: State College; locality: Park Forest Village Neighbourhood; decimalLatitude: 40.806; decimalLongitude: -77.913; geodeticDatum: WGS84; **Identification:** identifiedBy: Denise C. Montelongo; **Event:** samplingProtocol: hand collected; eventDate: 2020-07-07; fieldNumber: DCM2020-243; **Record Level:** institutionCode: INHS; basisOfRecord: PreservedSpecimen
- **Type status:** Paratype. **Occurrence:** catalogNumber: PSUC_FEM_79465; occurrenceRemarks: emerged 14-17.viii.2020 | Dropped Gall; recordedBy: Andrew R. Deans; individualCount: 1; sex: male; lifeStage: adult; occurrenceID: D1EDA88E-F1D0-572E-B7AC-C1491188CF03; **Taxon:** scientificName: Synergus nigrus; order: Hymenoptera; family: Cynipidae; **Location:** stateProvince: Pennsylvania; county: Centre; municipality: Ferguson Township; locality: Pine Grove Mills; decimalLatitude: 40.731; decimalLongitude: -77.889; geodeticDatum: WGS84; **Identification:** identifiedBy: Denise C. Montelongo; **Event:** samplingProtocol: hand collected; eventDate: 2020-07-27; fieldNumber: DCM2020-358; **Record Level:** institutionCode: USNM; basisOfRecord: PreservedSpecimen
- **Type status:** Paratype. **Occurrence:** catalogNumber: PSUC_FEM_79466; occurrenceRemarks: emerged 30.vii-2.viii.2020 | Dropped Gall; recordedBy: Laura Porturas; individualCount: 1; sex: female; lifeStage: adult; occurrenceID: 3686024B-3E2B-5534-9996-F4185609F2B5; **Taxon:** scientificName: Synergus nigrus; order: Hymenoptera; family: Cynipidae; **Location:** stateProvince: Pennsylvania; county: Centre; municipality: State College; locality: Park Forest Village Neighbourhood; decimalLatitude: 40.806; decimalLongitude: -77.913; geodeticDatum: WGS84; **Identification:** identifiedBy: Denise C. Montelongo; **Event:** samplingProtocol: hand collected; eventDate: 2020-07-07; fieldNumber: DCM2020-244; **Record Level:** institutionCode: USNM; basisOfRecord: PreservedSpecimen
- **Type status:** Paratype. **Occurrence:** catalogNumber: PSUC_FEM_79467; occurrenceRemarks: emerged 30.vii-2.viii.2020 | Dropped Gall; recordedBy: Laura Porturas; individualCount: 1; sex: female; lifeStage: adult; occurrenceID: 929A947F-8D15-559E-B217-20823F9BF349; **Taxon:** scientificName: Synergus nigrus; order: Hymenoptera; family: Cynipidae; **Location:** stateProvince: Pennsylvania; county: Centre; municipality: State College; locality: Park Forest Village Neighbourhood; decimalLatitude: 40.806; decimalLongitude: -77.913; geodeticDatum: WGS84; **Identification:** identifiedBy: Denise C. Montelongo; **Event:** samplingProtocol: hand collected; eventDate: 2020-07-07; fieldNumber: DCM2020-233; **Record Level:** institutionCode: USNM; basisOfRecord: PreservedSpecimen
- **Type status:** Paratype. **Occurrence:** catalogNumber: PSUC_FEM_79468; occurrenceRemarks: emerged 27-30.vii.2020 | Dropped Gall; recordedBy: Laura Porturas; individualCount: 1; sex: female; lifeStage: adult; occurrenceID: BE968F60-F378-5F08-9BCB-5B7F17042B28; **Taxon:** scientificName: Synergus nigrus; order: Hymenoptera; family: Cynipidae; **Location:** stateProvince: Pennsylvania; county: Centre; municipality: State College; locality: Park Forest Village Neighbourhood; decimalLatitude: 40.806; decimalLongitude: -77.913; geodeticDatum: WGS84; **Identification:** identifiedBy: Denise C. Montelongo; **Event:** samplingProtocol: hand collected; eventDate: 2020-07-07; fieldNumber: DCM2020-334; **Record Level:** institutionCode: USNM; basisOfRecord: PreservedSpecimen
- **Type status:** Paratype. **Occurrence:** catalogNumber: PSUC_FEM_79469; occurrenceRemarks: emerged 2-4.viii.2020 | Dropped Gall; recordedBy: Laura Porturas; individualCount: 1; sex: female; lifeStage: adult; occurrenceID: ED83F6DB-8962-5136-8554-B3BE50C6C066; **Taxon:** scientificName: Synergus nigrus; order: Hymenoptera; family: Cynipidae; **Location:** stateProvince: Pennsylvania; county: Centre; municipality: State College; locality: Park Forest Village Neighbourhood; decimalLatitude: 40.806; decimalLongitude: -77.913; geodeticDatum: WGS84; **Identification:** identifiedBy: Denise C. Montelongo; **Event:** samplingProtocol: hand collected; eventDate: 2020-07-07; fieldNumber: DCM2020-237; **Record Level:** institutionCode: USNM; basisOfRecord: PreservedSpecimen

#### Description

**Female body length**: 2.5–3.4 mm (n = 13 specimens examined).

**Figure 1.**
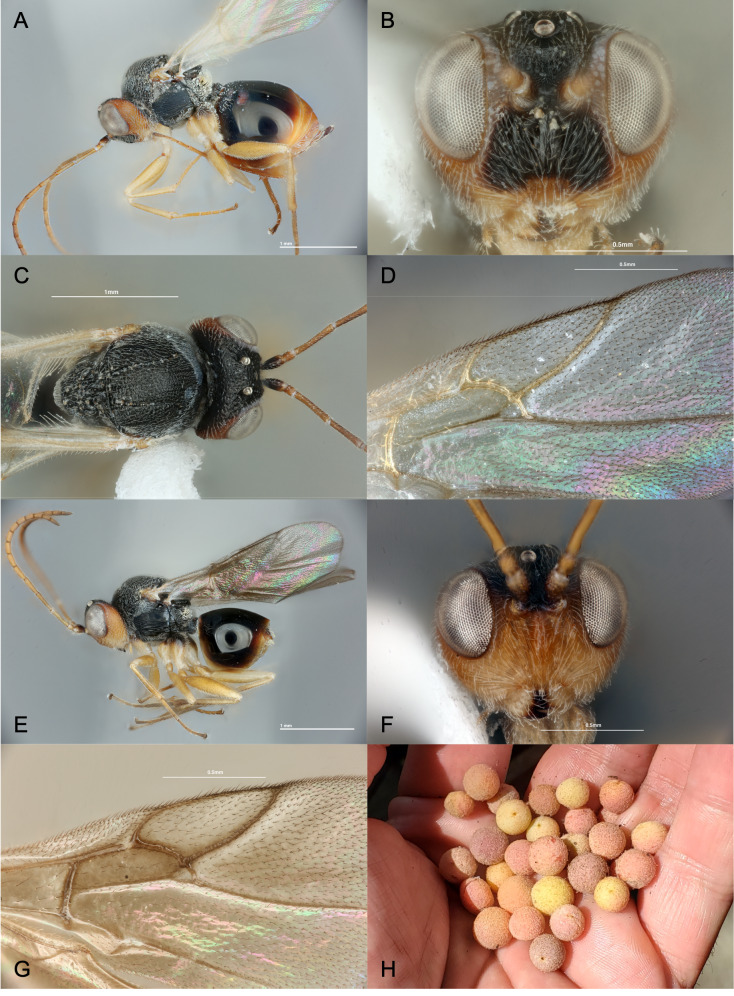
*Synergus nigrus* Montelongo & Nastasi, sp. nov., holotype female (PSUC_FEM_79450). **A** lateral view; **B** head in frontal view; **C** mesosoma in dorsal view; **D** radial cell of fore wing. Male: **E** lateral view; **F** head in frontal view; **G** radial cell of fore-wing. **H** Gall of host, *Philonix nigra*. Scale bars: 1 mm (**A, C, E**); 0.5 mm (**B, D, F, G**). Photograph (**A-G**) (CC BY) Louis F. Nastasi and (**H**) (CC BY) Andrew R. Deans.

**Colour**: Body black and yellow. Antennae mostly yellow, gradually darkening distally and with scape dark brown to black. Head black dorsally and amber ventrally the toruli. Lower face and malar space yellow (Fig. 1F); sometimes lower face predominantly black (Fig. 1B). Mesosoma entirely black, tegulae amber. Coxae, femora and tibiae yellow, although metacoxae with black maculation posterodorsally, extending more or less one-third of the length of the coxa from the base. Tarsi light brown to black. Wings hyaline, veins yellow, darkened distally. Metasoma primarily black with amber inflections on edges of syntergum and entirety of following terga; with a conspicuous brownish spot anterolaterally; hypopygium amber.

**Head**: Head shape trapezoidal in anterior view, slightly wider than high, gena not broadened posterior to eye. Face moderately pubescent. Facial radiating striae complete, dense and regular, radiating from clypeus and reaching eyes and toruli; medial striae present. Clypeus indistinct. Malar space 0.5× as long as compound eye. Anterior tentorial pits visible; pleurostomal and epistomal sulci absent. Transfacial line about as long as height of eye. Toruli situated slightly ventral of the midline of eyes; distance between torulus and eye equal to diameter of toruli; distance between toruli equal to diameter of either torulus. Frons coriaceous, with small piliferous punctures; frontal carinae wide and strongly curved, reaching from each lateral ocellus to dorsal margin of each torulus. Head in dorsal view about twice as wide as long. Vertex coriaceous, with some conspicuous punctures. POL:OOL:LOL:DLO approximately 10:7:5:4. Occiput coriaceous, with scattered setigenous punctation.

**Figure 2.**
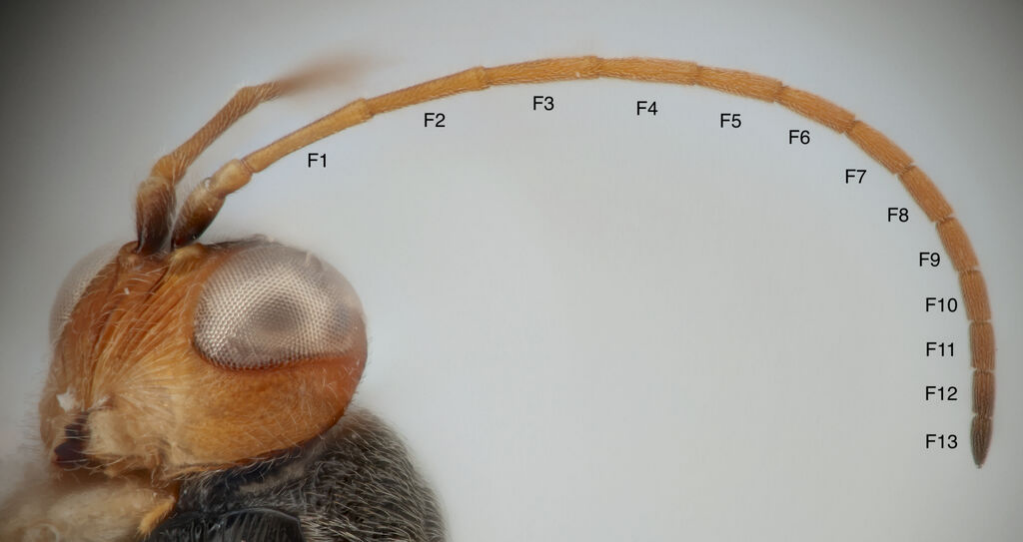
*Synergus nigrus* antenna, exhibiting 13 flagellomeres (F1–13) (Specimen PSUC_FEM_79452).

**Antenna**: Antennae filiform, not broadened apically; pubescence dense and short; scape and pedicel with longer pubescence. Scape plus pedicel slightly shorter than F1. Pedicel about 1.4× as long as wide. Flagellum subdivided into 13 flagellomeres (Fig. 2). F1 about 7.8× as long as wide; F1 longer than F2, the following subsegments progressively shorter. Apical flagellomere about 2.7× as long as wide.

**Mesosoma**: About 1.2× as long as high in lateral view including nucha, with moderately dense short pilosity. Pronotal plate defined anteriorly, with lateral margins short and ending far from anterior margin of pronotum. Lateral pronotum carinate-rugose. Pronotum rounded in dorsal view. Mesoscutum about 1.2× wide as long, coriaceous with weak transverse elements not forming true carinae; anterior parallel lines weakly impressed, reaching about 1/3 of the mesoscutal length. Notauli complete. Median mesoscutal line short, just appearing as a small incision. Parapsidal lines shallow, but distinct. Mesoscutellum rounded, about as long as wide, wrinkled, interspaces coriaceous; circumscutellar carina visible; scutellar foveae ovate, smooth, well defined and impressed and separated by a narrow carina. Mesopleuron finely, regularly and densely striate with a smooth patch under the speculum; slightly pubescent basally. Metapleural sulcus reaches 3⁄4 parts of mesopleuron height. Propodeum moderately densely pilose; propodeal carinae narrow, slightly curved throughout and convergent both dorsally and ventrally. Nucha distinctly latitudinally striate.

**Legs**: Tarsal claw bidentate, with a blunt basal tooth.

**Wings**: Fore wing longer than body length; pubescent with short marginal setae. Marginal cell closed and 2.8× as long as wide. Basal cell with sparse setae. Areolet closed.

**Metasoma**: About as long as head plus mesosoma, almost 1.2× as long as high in lateral view. First metasomal segment sulcate dorsally and laterally. Syntergum smooth, with an anterolateral pubescence composed of a few setae, sometimes just a posterior dorsal patch. Following segments conspicuously punctate throughout. Hypopygium punctate; hypopygial spine almost 2.0× as long as high with a few lateral setae and with apical setae present.

**Male**: Same as female except for the following: Body length 1.9–2.9 mm (n = 5). Lower face yellow (Fig. 1F). Syntergum with a band of micropunctures. Wings smoky with brown veins. POL:OOL:LOL:DLO approximately 10:6:5:4.

#### Diagnosis

Mesopleuron conspicuously sculptured throughout, except for smooth patch ventral of speculum; female antennae with 15 antennomeres; dorsal metacoxa with conspicuous dark spot; similar to *S. villosus* in colouration (Fig. 1A), but females sometimes with black markings on the lower face (Fig. 1B); F1 longer than F2 (cf. *S. villosus*, where F1 shorter than F2); male with slightly infuscated wings; females wings hyaline. The distinctive wing colourations could enable separation of male *S. nigrus* should males of *S. villosus* be found to have hyaline wings. Male *S. villosus* are currently unknown (Lobato-Vila and Pujade-Villar 2021). The six known species in the Nearctic to have females with 15 antennomeres (13 flagellomeres; *S. atripennis*, *S. dorsalis*, *S. kinseyi*, *S. ochereus*, *S. quercuslana* and *S. villosus*) all have completely sculptured mesopleura.

When utilising the Lobato-Vila and Pujade-Villar (2021) *Synergus* key, female *S. nigrus* keys to couplet 60, where *S. nigrus* is easily differentiated from *S. quercuslana* by F1 being longer than F2. Males key to couplet 26, where *S. nigrus* can be differentiated from *S. atripennis* by the characters given above.

Although the new species is morphologically similar to *S. villosus*, it has diagnostic features different from all known *Synergus* (see taxon treatment below). These did not match any other known *Synergus* species and its combination of traits did not align with those of other genera.

#### Etymology

The specific epithet *nigrus* is derived from the species’ host gall *Philonix nigra*.

#### Distribution

Collected in Canada: Manitoba (deWaard et al. 2019) and reared in USA: Kansas (Ward et al. 2020) and Pennsylvania. The species likely spans a broad eastern Nearctic distribution, similar to that of *Philonix* (GBIF Secretariat 2023).

#### Biology

Reared from galls of the asexual generation of *P. nigra* on *Q. alba* , emerging between late July to early September in Pennsylvania. A male was previously reared from *P. nigra* on *Quercus macrocarpa* (Ward et al. 2020), where it was given a placeholder name of *Synergus* sp. #2. The voucher of *Synergus* sp. #2 was destructively sampled for DNA barcoding; however, an image of the specimen was examined and its identity as *Synergus nigrus* was confirmed.

## Analysis

### Diagnostic results

**Figure 3.**
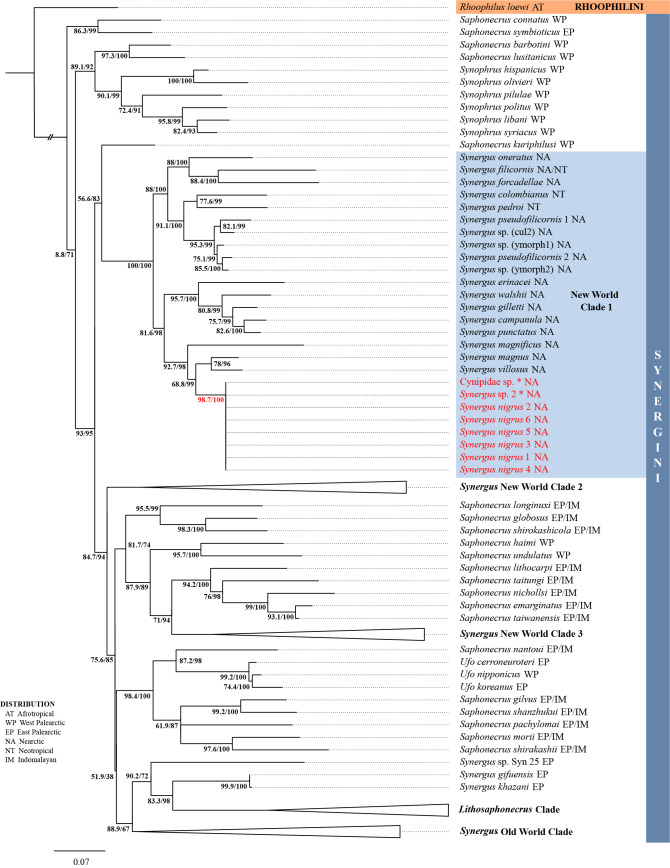
Maximum Likelihood (ML) tree resulting from the analysis of COI, across Synergini and including the newly-added specimens of *Synergus nigrus*. SH-aLRT support (%) / ultrafast bootstrap support (%) values are depicted next to respective nodes. * Sequences on GenBank matching *Synergus nigrus* sequences.

This new species resolves as New World Clade 1 (Fig. 3) (SH-aLRT = 98.7, UFboot = 100) as a sister lineage to *Synergus magnus* and *Synergus villosus* (Suppl. material 3). The genetic similarity of COI for the six specimens (0% difference between them; genetic distances calculated in Geneious Prime 2021.1 (GraphPad Software, LLC, Boston, Massachusetts) with the Tamura-Nei distance model) and morphological homogeneity strongly suggest they are conspecific. Furthermore, the two unidentified sequences included from GenBank were 100% matches (Cynipidae sp. deWaard et al. (2019); *Synergus* sp. 2 Ward et al. (2020)), suggesting these are also conspecific with this new species.

## Discussion

Here, we describe a new species of inquilinous gall wasp, *Synergus nigrus*. This inquiline has, thus far, only been found in *P. nigra* galls on *Q. alba*, being reared by us and by Ward et al. (2020) (*Synergus* sp. #2). Three other species of *Synergus*, in more distant lineages, have independently evolved to attack *P. nigra* , suggesting this galler is especially susceptible to attack by *Synergus* (Ward et al. 2020).

The prevailing concept of all *Synergus* with a partially smooth mesopleuron having 14 antennomeres is now baseless, with the description of *S. nigrus* (Lobato-Vila and Pujade-Villar 2017, Lobato-Vila et al. 2020a). *Synergus villosus* and now *S. nigrus* are the only species of their genus with 15 antennomeres represented in genetic libraries, increasing their ease for future study (Lobato-Vila et al. 2019, Lobato-Vila et al. 2020b, Lobato-Vila and Pujade-Villar 2021).

The intraspecific morphological diversity in *Synergus* species makes species delimitation difficult, since some species demonstrate morphological variance while others do not, raising the idea that *Synergus* is plagued by widespread cryptic species. Thus, our findings add support to Ward et al. (2020) in their backing for a taxonomic revision of the Nearctic *Synergus* that incorporates molecular, morphological and ecological data.

## Supplementary Material

XML Treatment for Synergus nigrus

2C00AEBD-DAF8-50F0-801E-25637D67D3BA 10.3897/BDJ.14.e170671.suppl1 Supplementary material 1 GenBank accession numbers Data type genetic Brief description Accession numbers for DNA sequences used in our analyses. File: oo_1399974.csv https://binary.pensoft.net/file/1399974 Montelongo, Denise C.

BF0108BD-3D3F-5EF4-BEF9-62E53BB75232 10.3897/BDJ.14.e170671.suppl2 Supplementary material 2 Table S1.2 Morphological terms Data type morphological terminology Brief description Table of terms used in this manuscript to describe phenotypic characters, with definitions and references to sources. File: oo_1399976.docx https://binary.pensoft.net/file/1399976 Montelongo, Denise C. and Nastasi, Louis F.

90ACFB9B-7666-544D-BDE6-7F4833AB4512 10.3897/BDJ.14.e170671.suppl3 Supplementary material 3 COI barcode tree Data type phylogeny Brief description Complete phylogeny of all COI sequences analysed. File: oo_1399979.svg https://binary.pensoft.net/file/1399979 Montelongo, Denise C.
