# Supplementary material for: Synergus nigrus (Hymenoptera, Cynipidae, Synergini), a new inquiline species reared from galls of Philonix nigra Gillette, 1889 (Cynipidae, Cynipini)
Source: Biodivers Data J. 2026 Mar 27;14:e170671. doi: 10.3897/BDJ.14.e170671 (PMC13049454; doi:10.3897/BDJ.14.e170671)
Supplement: Supplementary material 2 — Table S1.2 Morphological terms [file bdj-14-e170671-s002.docx]

Table S**1.2**: URI table showing terms used, their definition and source.

|  |
| --- |

| **Terms** | **Definition** | **URI or Concept ID + ontology URL** |
| --- | --- | --- |
| antenna | The appendage that is composed of ringlike sclerites and the anatomical structures encircled by these sclerites and that is articulated with the cranium. | <http://purl.obolibrary.org/obo/HAO_0000101> |
| area | The anatomical structure that is delimited by material or immaterial anatomical entities. | <http://purl.obolibrary.org/obo/HAO_0000146> |
| areolet | The wing cell of the fore wing that is minute, round and is delimited by the radio-medial cross veins. | <http://purl.obolibrary.org/obo/HAO_0000147> |
| basal cell | Fore wing cell enclosed by R+Sc, M, and M+Cu_1_. | Melika and Abrahamson (2000) |
| body | The anatomical cluster that is composed of the whole organism but which excludes the antennae, legs and wings | <http://purl.obolibrary.org/obo/HAO_0000182> |
| carina | The process that is elongate and external. | <http://purl.obolibrary.org/obo/HAO_0000188> |
| circumscutellar carina | The carina that encompasses a horizontal, median, rounded area of the mesoscutellar disc. | <http://purl.obolibrary.org/obo/HAO_0001943> |
| clypeus | The area that corresponds to the site of origin of the clypeo-epipharyngeal muscle. | <http://purl.obolibrary.org/obo/HAO_0000212> |
| coxa | The leg segment that is connected to the body and to the trochanter via conjunctivae and muscles. | <http://purl.obolibrary.org/obo/HAO_0000228> |
| eye | The compound organ that is composed of ommatidia. | <http://purl.obolibrary.org/obo/HAO_0000217> |
| femora | The leg segment that is distal to the trochanter and proximal to the tibia. | <http://purl.obolibrary.org/obo/HAO_0000327> |
| flagellum | The anatomical cluster composed of flagellomeres. | <http://purl.obolibrary.org/obo/HAO_0000343> |
| fore wing | The wing that is located on the mesothorax. | <http://purl.obolibrary.org/obo/HAO_0000351> |
| frons | The area that is located dorsally of the ventral margin of the antennal rim and ventrally of the anterior ocellus medial to the inner margins of the eye and malar line. | <http://purl.obolibrary.org/obo/HAO_0001044> |
| gena | The area that is delimited by the intersection of the interorbital plane, the margin of the compound eye, the margin of the oral foramen, the occipital carina and the malar sulcus. | <http://purl.obolibrary.org/obo/HAO_0000371> |
| head | The tagma that is located anterior to the thorax. | <http://purl.obolibrary.org/obo/HAO_0000397> |
| hypopygium | The abdominal sternum that is the posteriormost visible sclerite located ventrally in the abdomen. | <http://purl.obolibrary.org/obo/HAO_0000410> |
| leg | The anatomical cluster that is composed of the coxa and all distal leg segments and is connected to the pectus. | <http://purl.obolibrary.org/obo/HAO_0000494> |
| lower face | The area that is limited dorsally by the ventral margin of the antennal foramen laterally by the malar sulcus and ventrally by the oral foramen. | <http://purl.obolibrary.org/obo/HAO_0000502> |
| malar space | The anatomical line that is the shortest between the lower orbit and the ventrolateral margin of the cranium. | <http://purl.obolibrary.org/obo/HAO_0000503> |
| margin | The line that delimits the periphery of an area. | <http://purl.obolibrary.org/obo/HAO_0000510> |
| marginal cell | Fore wing cell enclosed by R1, 2r, Rs and wing margin. | Nastasi et al. 2024a, b |
| marginal cell ratio | Coefficient between its length (from the confluence point of the R1 and the 2r veins to the end of the Rs vein) and its width (from the upper margin of the fore wing to more or less the beginning of the Rs vein) | Nastasi et al. 2024a, b |
| mesopleuron | The area that is located laterally of the mesodiscrimen. | <http://purl.obolibrary.org/obo/HAO_0000566> |
| mesoscutellum | The scutellum that is located on the mesonotum. | <http://purl.obolibrary.org/obo/HAO_0000574> |
| mesoscutum | The scutum that is located on the mesonotum. | <http://purl.obolibrary.org/obo/HAO_0000575> |
| metacoxa | The coxa that is located on the hind leg. | <http://purl.obolibrary.org/obo/HAO_0000587> |
| metasoma | The tagma that is connected anteriorly to the metapectal-propodeal complex at the propodeal foramen and consists of abdominal segments. | <http://purl.obolibrary.org/obo/HAO_0000626> |
| notauli | The line that extends submedially along the mesoscutum and corresponds to the median border of the site of origin of the first mesopleuro-mesonotal muscle. | <http://purl.obolibrary.org/obo/HAO_0000647> |
| nucha | The area that is raised and surrounds the propodeal foramen. | <http://purl.obolibrary.org/obo/HAO_0000651> |
| occiput | The area that is concave and surrounds the postocciput. | <http://purl.obolibrary.org/obo/HAO_0000658> |
| ocellus | The multi-tissue structure that is located on the top of the head, composed of the corneal lens, pigment cell, rhabdoms and synaptic plexus. | <http://purl.obolibrary.org/obo/HAO_0000661> |
| pedicel | The antennal segment that is connected proximally to the scape and distally to the flagellum. | <http://purl.obolibrary.org/obo/HAO_0000706> |
| plate | The area of the integument where the cuticle is well sclerotized with thick exocuticle. | <http://purl.obolibrary.org/obo/HAO_0000909> |
| pronotum | The notum that is located in the prothorax. | <http://purl.obolibrary.org/obo/HAO_0000853> |
| scape | The antennal segment that is proximal to the pedicel and is connected to the head via the radicle. | <http://purl.obolibrary.org/obo/HAO_0000908> |
| scutellar fovea | The depression that is medially located on the scutoscutellar suture. | <http://purl.obolibrary.org/obo/HAO_0000916> |
| segment | The anatomical cluster that is connected to other segments via conjunctivae and muscles and is delimited by its sclerites. | <http://purl.obolibrary.org/obo/HAO_0000929> |
| speculum | The area that is located dorsolaterally on the mesosoma, is delimited posteriorly by the mesepimeral ridge and corresponds to the site of origin of the anterior mesopleuro-mesofurcal muscle. | <http://purl.obolibrary.org/obo/HAO_0000944> |
| sulcus | The groove that corresponds to a ridge. | <http://purl.obolibrary.org/obo/HAO_0000978> |
| syntergum | The sclerite that is composed of fused abdominal terga. | <http://purl.obolibrary.org/obo/HAO_0000987> |
| tarsal claw | The spur that is curved and projects from the apex of the last tarsal segment on either side of the arolium of the pretarsus. | <http://purl.obolibrary.org/obo/HAO_0000989> |
| tarsus | The leg segment that is apical to the tibia. | <http://purl.obolibrary.org/obo/HAO_0000992> |
| tegula | The cuticular evagination that is located laterally of the preaxilla and obscures the anterior mesonoto-first axillary articulation and the mesopleuro-second axillary sclerite joints. | <http://purl.obolibrary.org/obo/HAO_0000993> |
| anterior tentorial pit | The tentorial pit that corresponds to the anterior end of the tentorium and partially delimits the clypeus. | <http://purl.obolibrary.org/obo/HAO_0000126> |
| tergite | The sclerite that is located on the tergum. | <http://purl.obolibrary.org/obo/HAO_0001005> |
| tibia | The leg segment that is proximal to the tarsus and distal to the femur. | <http://purl.obolibrary.org/obo/HAO_0001017> |
| torulus | The rim that surrounds the antennal foramen. | <http://purl.obolibrary.org/obo/HAO_0000103> |
| transfacial line | The distance between inner margins of eyes measured across toruli. | Lobato-Vila and Pujade-Villar (2021) |
| veins | The area that is linear and sclerotized and acts as support for the wing membrane. | <http://purl.obolibrary.org/obo/HAO_0001095> |
| vertex | The area that is delimited by the intersection of the margin of the compound eyes, the interorbital plane, and the anatomical line that is tangential to the point on the margin of the anterior ocellus which defines the minimum distance between the anterior ocellus and the oral foramen. | <http://purl.obolibrary.org/obo/HAO_0001077> |
| wing | The area that is located in between the notum and the pleuron and is composed of the wing base and wing blade. | <http://purl.obolibrary.org/obo/HAO_0001089> |
